# Supplementary material for: Higher socioeconomic status does not predict decreased prosocial behavior in a field experiment
Source: Nat Commun. 2021 Jul 12;12:4266. doi: 10.1038/s41467-021-24519-5 (PMC8275767; doi:10.1038/s41467-021-24519-5)
Supplement: Supplementary file 1 — Supplementary Information [file 41467_2021_24519_MOESM1_ESM.pdf]

SUPPLEMENTARY INFORMATION FOR: Higher socioeconomic status does not predict decreased prosocial behavior in a field experiment

Andreoni, Nikiforakis and Stoop, 2021

June 13, 2021

Supplementary Note 1: Details on the selection of high and low SES households

All households were located in the same medium-sized city in the Netherlands. Households were randomly selected through a two-step process. In the first step, to ensure that we selected either high or low SES individuals to participate in our experiment, we compiled lists of high and low SES households following the procedure detailed below. In the second step, we randomly selected households from these lists for participation.

Our first concern was to compile a list of high and low SES households. CBS Netherlands was willing to provide us with such data for each household *after* the experiment was conducted. This way, they could encrypt the data, guaranteeing the privacy and anonymity of all participants. For safety and privacy concerns, no institution was willing to share with us a list that would be linking individual addresses with household wealth, *prior* to data collection. For this reason, we had to compile our own list of households to select from using a measure that would be highly correlated with wealth. We used the property value of one's house for high SES individuals, and the rental price for the low SES individuals.

Selecting low SES households

The city has several social-housing corporations. For one of these, the explicit purpose is to rent out apartments to the lowest SES people in the city. The social-housing corporation was kind enough to share with us a list with the addresses of their cheapest apartments, from which we selected 152 households. As this number did not suffice for our study, we selected additional apartments by using the website of the same corporation in which apartments are advertised for rent. In particular, we selected apartments in listed buildings, but not the apartments that were for rent.

After compiling the list of low SES households, we randomly selected 225 of them to participate in our experiment (180 used in our main experiment and 45 used in the "Low SES Joost" treatment), provided that two conditions were met. First, most apartment buildings have four stories and share entrances with eight apartments. Although an official from the social-housing corporation informed us that residents have little contact with each other, we decided to limit misdeliveries to three mailboxes per entrance to minimize communication between households. We randomly selected 159 households sharing an entrance with two other apartments in the same building, and 66 households that do not share an entrance. We find no economically or statistically significant differences across these two groups. Households sharing entrances returned 38.4% of misdelivered envelopes while household not sharing entrances in our sample returned 47.0% (N=225, $p = 0.24$, two-tailed, Fisher-exact).

The second condition was that the family living in a selected household is native Dutch. The reason is that the ethnicity of the sender and receiver can influence pro-social behavior

[1, 2, 3, 4]. This could be a problem as non-natives in the Netherlands tend to have a greater likelihood of having a lower SES. To avoid this confound, we took pictures of all family-name signs on doorbells and ensured that all apartments in our sample were likely to be native Dutch. Using data from CBS Netherlands, we can control for the effect of any remaining non-Dutch households.

Selecting high SES households

To compile a list of high SES households, we started by consulting www.Funda.nl, a website in the Netherlands that advertises houses for sale. Our goal was to identify neighborhoods and streets where wealthy individuals live. To do this, we found all houses with a sale price of at least €750,000. All houses on the same street as the identified houses were included on our list of high SES households, excluding the houses that were up for sale. Once this procedure was done, we consulted www.Postcode.nl. This website contains information on all houses in the city in which our experiment was conducted such as the postal code, house number, and surface. We used this website to include on our list all houses that were not for sale, but had a comparable surface as those that were for sale.

Once the list was compiled, we randomly selected high SES households to deliver the misdelivered letter. To minimize effects of communication between subjects, we excluded houses that were close to each other. A total of 100 houses in our sample have no neighboring house that is selected for any of the core treatments. The other 80 houses do have at least one neighbor that is a subject in one of the core treatments. (Almost all of these houses are villas with a large surface and driveways that are far removed from the driveway of the neighbor.) Differences in return rates between houses with a neighbor (76.3%), or without neighbour (85.0%) are insignificant (Fisher exact test, $p = 0.18$).

Supplementary Note 2: Household-level data and randomization check

We collected information from CBS Netherlands on the households in our sample for three purposes: (i) to ensure that we correctly identified high and low SES households, (ii) to ensure that our randomization of households into treatments was successful, and (iii) to check the robustness of our experimental findings when adding additional controls. An overview of this can be found in Supplementary Table 1.

Supplementary Table 1 reveals that we have successfully identified high and low SES households. Specifically, we see high SES individuals have over 4 times the income of low SES individuals, and over 90 times the wealth. The randomization into treatments was also successful. Using Mann-Whitney tests, we cannot reject the hypothesis of an equal distribution of disposable income among high SES individuals ($p = 0.53, N_1 = 88, N_2 = 83$)

Supplementary Table 1 Observable characteristics of high and low SES households; entries are means

Treatment	Disposable Income	Wealth	HH Age	Bene-fits	HH Size	Pension	HH Foreign	Distance Joost	Distance Mailbox	Density
				Panel A. High SES Households						
BTC	87,482	2,570,564	56.18	0.09	2.78	0.44	0.00	4.04	0.35	0.44
Cash	81,533	2,422,694	56.69	0.07	2.76	0.42	0.00	4.28	0.35	0.42
Average	84,508	2,496,629	56.44	0.08	2.77	0.43	0.00	4.16	0.35	0.43
				Panel B. Low SES Households						
BTC	19,063	20,988	43.82	0.31	1.43	0.16	0.16	3.54	0.25	0.35
Cash	18,983	33,485	41.09	0.36	1.44	0.13	0.15	3.52	0.25	0.35
Average	19,023	27,237	42.45	0.33	1.43	0.14	0.15	3.53	0.25	0.35

and among low SES individuals ($p = 0.71, N_1 = 88, N_2 = 88$).[1] The same holds for wealth of high SES individuals ($p = 0.27$), but not for wealth of low SES individuals ($p = 0.05$). A similar analysis is done for all other variables in Supplementary Table 1, using Fisher Exact tests for dummy variables. All p-values are 0.23 or greater.

Supplementary Table 1 shows that high SES individuals differ from low SES individuals in more than just disposable income and wealth. High SES individuals, on average, are: older, live further from Joost and the nearest mailbox, have larger families, are less ethnically diverse, receive more pensions, but fewer benefits. We control for these in a regression analysis that is presented later on in the Supplementary Information. We note that, overall, we do not find a difference between high and low SES households when we compare households for which at least one member receives either a pension or benefits (implying that at least one family member is likely to be at home). The share of such households is similar (0.49 for high SES individuals and 0.45 for low SES individuals) and not significantly different (Fisher exact test, $N_1 = 180, N_2 = 180, p = 0.53$). This echoes a finding reported in [5] that there is no difference in the amount of leisure time between high SES individuals and the general population, in the Netherlands.

Definitions of socio-economic variables

CBS Netherlands provides administrative data on all citizens of the Netherlands. Data are available from various sources, and they can be merged on an individual or household level. To guarantee anonymity, CBS Netherlands encrypts each person and household by means of a code called RIN-address and RIN-person. CBS Netherlands has encrypted the addresses in the database that we provided them, by adding the variable RIN-address. Then, we merged different databases of CBS Netherlands by using the RIN-address variable. Supplementary Table 2 gives an overview of the different databases that we used.

[1] The number of observations per treatment was 90 in all cases. CBS Netherlands was unable to provide us with information on certain variables in a small number of cases, as noted.

Supplementary Table 2 Overview of the databases used from CBS Netherlands.

Database	Version	Variables used
Marital Status administration	2013, actual	RIN-address
'GBA Burgerlijkestaatbus'		RIN-person
Integral Household Income	2013, actual	Disposable household income
'Integraal Huishoudens Inkomen'		Household size
Integral Wealth Database	2013, actual	Household Wealth
'Integraal Vermogensbestand'		
Municipality Persons Administration	2013, actual	Age
'GBApersoonstab'		Country of birth
Social Economic Category	2013, actual	Person receives:
'SECM'		Pension
		Unemployment benefits
		Social welfare
		Social security
		Disability insurance

CBS Netherlands has the following definitions of the variables that are not self-explanatory.

Income definitions:
Primary income consists of yearly income from labor (gross wages, salary, tantieme, compensation for labor not from employment), income from own company, and income from equity (interest, dividend, exploitation of real-estate).
Gross income consists of Primary income + received transfers (i.e. unemployment benefits, pension, etc.).
Disposable income consists of Gross income − income transfers (e.g. alimony) − premium income insurance − disability/illness insurance − tax on income and wealth.

Wealth definitions:
Wealth consists of the possessions − debts
Possessions consist of bank balance, securities, value of own house, 'other' real estate, 'other' possessions (i.e. cash, rented real estate, venture capital)
Debts consist of the mortgage on own house, 'other' debts to finance consumption goods, shares/bonds, second house.

Supplementary Note 3: On the envelopes and their content

The letters misdelivered in the experiment contained either a banknote of €5 or €20, or a bank-transfer card (BTC) of the same value. The analysis presented at the paper pooled the data from the different amounts. Here, we present the disaggregated analysis. Let BTCx and Ex denote the treatment with $x \in \{5, 20\}$. Return rates by treatment can be seen in Supplementary Fig. 1.

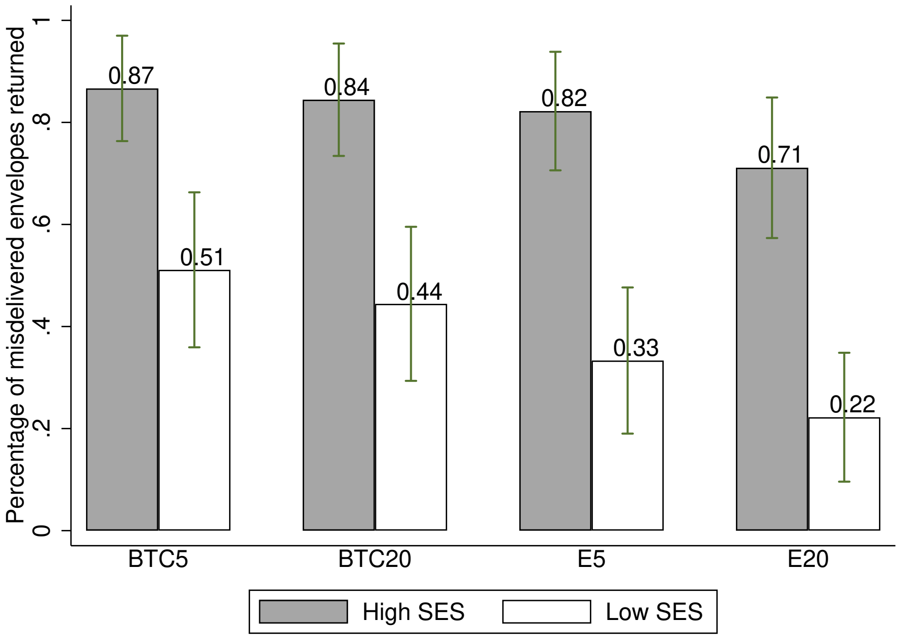

Supplementary Figure 1 Percentage of envelopes returned with 95-percent confidence intervals ($N = 45$ for each bar). Cash refers to the treatment in which the envelope contains a banknote of €5 or €20. BTC refers to the treatment in which the envelope contains a bank-transfer card of €5 or €20.

For high SES households, the return rate decreases insignificantly from 87% in BTC5 to 84% in BTC20 ($N = 90$, $p = 1.00$, two-tailed, Fisher-exact). For low SES households, the return rate decreases from 51% in BTC5 to 44% in BTC20, but the difference is again insignificant ($N = 90$, $p = 0.67$, two-tailed, Fisher-exact). The difference in return rates between E5 and E20 is insignificant both for high SES individuals (82% vs. 71%; $N = 90$, $p = 0.32$, two-tailed, Fisher-exact) and low SES individuals (33% vs. 22%; $N = 90$, $p = 0.35$, two-tailed, Fisher-exact). Importantly, we find that the high SES group is more likely to return a misdelivered letter in all four experimental conditions ($N = 90$, $p < 0.01$, two-tailed, Fisher-exact, for all conditions).

The content of the letter is visible through the use of semi-transparent envelopes (see Supplementary Figure 2). This raises the following question: Should one be concerned that the use of such envelopes affects our conclusions? One should indeed be concerned if two conditions are satisfied: (i) the envelopes make individuals suspicious, and (ii) suspicion affects differentially high and low SES individuals. With regards to (i), we first note that semi-transparent envelopes are not uncommon in the Netherlands, as are misdelivered letters. The key question then for answering (i) is indeed the following: Who sends money in a semi-transparent envelope? Most people understand that some people do extraordinary things, i.e., things that a large majority of people would not do. As long as there is a reasonable

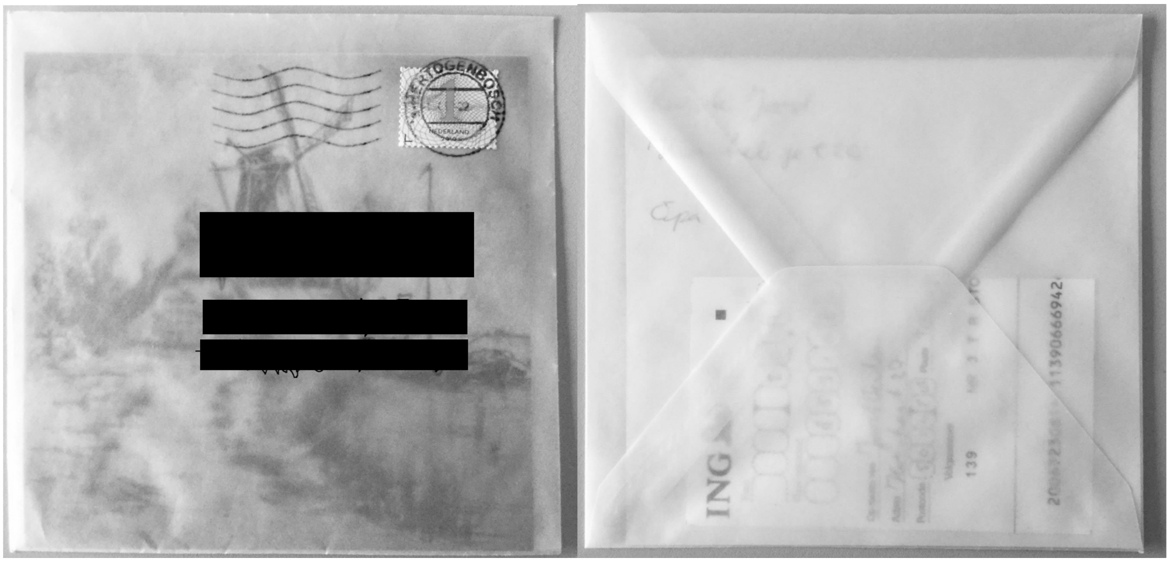

Supplementary Figure 2 An example of the front and the back of the envelopes used in the experiment. All envelopes included a postcard with the following handwritten message: “Dear Joost, here is €5 (or €20) for you. — Your grandfather.” The experimental treatments varied whether the amount was sent as a bank-transfer card (pictured on the right) or as a banknote.

possibility that someone could do X, there is no reason to be suspicious. This is why we decided to have the sender be a grandfather; a person that may recall more innocent times and be unaware of the dangers of sending money via post.

Anecdotal evidence from our experiment does not hint to participants becoming suspicious. Some individuals ($N = 16$) returned our envelopes after placing them in non-transparent A4-sized envelopes. All of them were accompanied by letters explaining that the mailman made a mistake (and usually added that Joost should advice his grandfather not to send money via post). None of these letters suggested that those returning the envelopes were suspicious. Still, in light of the above, even if some people became suspicious, we would expect them to be a small fraction of our sample. Therefore, we have no reason to expect that they would have a noticeable effect on our estimates, even if we had reasons to expect low SES individuals and high SES individuals to be differentially affected - which we do not.

Supplementary Note 4: Robustness checks

In this section, we present additional robustness checks by extending our regression analysis (see Table 1) to control for a number of variables obtained from CBS Netherlands. Supplementary Table 3 shows linear probability models. These controls are: (i) “Density” which measures the ratio of houses in a street participating in our experiment over the total houses in a street; (ii) “HH Foreign” is a dummy variable taking the value one if all adult members of a household are non-Dutch; (iii) “Benefits” is a dummy variable taking value one if at least

one adult member of the household receives unemployment benefits, social welfare, social security, or disability insurance; (iv) “Pension” is a dummy with value one if at least one adult member of the household receives pension; (v) “HH Size” measures the average number of people in a household, including children; (vi) “HH Age” measures the average age of all adults in a household. As can be seen, controlling for these additional variables does not affect our estimates appreciably or our conclusions.

Supplementary Note 5: Survey on the mailing habits of low SES individuals

In order to understand whether the lower return rates of low SES households might reflect differences in the use of postal services, we approached males and females on a street in the center of the city. Possible candidates were asked whether they were interested to participate in a short survey. If they responded positively, they were asked if they rented their home from a social housing corporation. If they responded negatively to this second question, then they were not interviewed. We set a target of 45 respondents, i.e., the same number as that of low SES households in each of our treatments.

Overall, 89 citizens responded that they were not interested to partake in our survey. 128 citizens were interested, but did not qualify. All 45 interviewed subjects answered all questions. The interviewer read the questions to respondents sequentially. The responses are given in parentheses in the questionnaire.

The questionnaire

We are conducting a survey regarding the use of postal services in different countries. We would be grateful if you could spent a couple of minutes answering a few questions.

1. How many letters do you usually send each week via post?

	More than 10 (2.2%)		Less than 5 (40.4%)
	Between 5 and 10 (4.4%)		I almost never send mail via post (53%)

2. How many letters do you usually receive each week in the post (excluding advertisements)?

	More than 10 (6.7%)		Less than 5 (62.2%)
	Between 5 and 10 (31.1%)		I do not have a mailbox (0.0%)

3. How often do you check your letterbox usually?

	Every day (75.5%)		Twice a week (4.4%)
	Once a week (2.2%)		Less than once a week (2.2%)

Supplementary Table 3 The determinants of returning envelopes

	(I)	(II)	(III)	(IV)	(V)	(VI)	(VII)	(VIII)	(IX)	(X)
High SES	0.43***	0.38***	0.31***	0.32***	0.30***	0.31***	0.30***	0.31***	0.28***	0.24***
	(0.04)	(0.07)	(0.07)	(0.08)	(0.07)	(0.07)	(0.07)	(0.07)	(0.08)	(0.08)
Cash		-0.20**	-0.20**	-0.20**	-0.20**	-0.20**	-0.20**	-0.20**	-0.19**	-0.19**
		(0.09)	(0.09)	(0.09)	(0.09)	(0.09)	(0.09)	(0.09)	(0.09)	(0.09)
Cash × High SES		0.11	0.11	0.11	0.12	0.11	0.11	0.11	0.11	0.10
		(0.09)	(0.10)	(0.10)	(0.10)	(0.10)	(0.09)	(0.10)	(0.09)	(0.10)
Week			-0.08**	-0.08***	-0.08**	-0.08**	-0.08**	-0.08***	-0.08***	-0.08**
			(0.03)	(0.03)	(0.03)	(0.03)	(0.03)	(0.03)	(0.03)	(0.03)
Week × High SES			0.08**	0.08*	0.08**	0.08**	0.08**	0.08**	0.08**	0.08**
			(0.04)	(0.04)	(0.04)	(0.04)	(0.04)	(0.04)	(0.04)	(0.04)
Distance Rec. House			-0.01	-0.01	-0.01	-0.01	-0.01	-0.01	-0.01	-0.01
			(0.01)	(0.01)	(0.01)	(0.01)	(0.01)	(0.01)	(0.01)	(0.01)
Distance Mailbox			-0.14**	-0.14**	-0.14**	-0.14**	-0.14**	-0.14**	-0.14**	-0.14**
			(0.05)	(0.05)	(0.05)	(0.05)	(0.05)	(0.05)	(0.05)	(0.06)
Density				-0.08						-0.05
				(0.14)						(0.14)
HH Foreign					-0.09					-0.10
					(0.09)					(0.09)
Benefits						-0.01				-0.01
						(0.06)				(0.06)
Pension							0.03			-0.02
							(0.06)			(0.09)
HH Size								-0.00		0.02
								(0.02)		(0.02)
HH Age									0.00	0.00
									(0.00)	(0.00)
Constant	0.38***	0.48***	0.64***	0.66***	0.65***	0.64***	0.63***	0.64***	0.54***	0.52***
	(0.03)	(0.06)	(0.08)	(0.09)	(0.08)	(0.08)	(0.08)	(0.08)	(0.10)	(0.13)
Observations	360	360	360	360	360	360	360	360	360	360
R-squared	0.1947	0.2196	0.2387	0.2390	0.2411	0.2387	0.2396	0.2387	0.2425	0.2466

Estimates from a linear probability model. The dependent variable is a dummy taking the value of 1 when an envelope is returned and 0 otherwise. "High SES" and "Cash" are dummy variables indicating whether an observation is associated with a high SES household or the Cash treatment, respectively. "Week" measures the number of weeks since the last payday. Distance Recipient's house (Distance mailbox) presents the distance from a subject's household to the recipient's house (the nearest mailbox). Standard errors are shown in parentheses (clustered at the street level). The results are virtually identical when we use a Probit specification. Reported results are from two-tailed tests. ***, **, * indicate significance at the .01, .05, and .10 levels, respectively.

4. Has the postal service ever misdelivered a letter in your letterbox that was meant to be delivered to a different address? YES/NO (100% yes)

5. If such a misdelivered letter were put in your letterbox, can you tell me some ways you can use so that the letter is properly delivered to the intended recipient? (Check all that they state.)

a) Drop it in a street mail box (88.9%)
b) Take it to the post office (24.4%)
c) Give it to a postal carrier (13.3%)
d) Hand deliver it myself (66.67%)
e) Put it in a new envelope and mail it (0.0%)
f) It can't be done (0.0%)
g) I don't know (0.0%).
h) Other: (31.1% of subjects came up with at least one alternative)

[*The following question was only asked to individuals who did not give (a) as an answer in question 5.*]
6. Suppose you were to simply drop the misdelivered envelope into a mailbox. What do you think the postal service would do with the envelope? (check all that they say)

a) Deliver it to the intended recipient (1 answer)
b) Return to the sender
c) Throw it away (1 answer)
d) I don't know (2 answers)

7. Can you tell me where the nearest mailbox is from your home? YES/NO (YES=91%)
If YES, about how long would it take you to walk there?_____ minutes (mean=3.2 minutes)

Supplementary Note 6: Survey on trusting institutions

To explore whether low SES individuals trust the public mail company less than high SES individuals, we chose to conduct a survey using Dutch students at Erasmus University Rotterdam. In total, we surveyed 140 students, but omit 7 observations from the analysis as they are from non-Dutch students. Students were given ample of space and time to fill out the survey. During the survey, students were forbidden to communicate with each other. Below, we present the survey instrument, as well as summary statistics in parentheses.

The questionnaire

Dear student,

We would like you to spend a couple of minutes to fill out this survey. After everyone is done, we will collect all forms. It is important to realize that there are no 'right' or 'wrong' answers, and that you are fully anonymous.

1. What is your age: ______ (average: 20.01)

2. What is your gender: (69.40% Male, 30.60% Female)

3. Where were you born? (100% Netherlands)

4. We are going to name a number of organizations in the Netherlands. For each one, could you tell us how much confidence you have in them: is it a great deal of confidence, quite a lot of confidence, not very much confidence, or none at all?

	A great deal	Quite a lot	Not very much	None at all	Average
The government	1	2	3	4	2.17
The police	1	2	3	4	2.02
The postal service	1	2	3	4	1.99
The press	1	2	3	4	2.68

5. Below is an income scale on which 1 indicates the lowest income group and 10 the highest income group in your country. We would like to know in what group the household of your parents is. Please, specify the appropriate number, counting all wages, salaries, pensions, and incomes that come in.

Lowest group							Highest group		
1	2	3	4	5	6	7	8	9	10
1%	1 %	3 %	7%	3%	15%	31%	27%	9%	3%

Supplementary Note 7: The Private Incentives treatment

This section includes the material for the additional treatment in which we explore the propensity of high and low SES households to return letters in the absence of prosocial concerns for doing so.

Participants received an official Erasmus University Rotterdam A4-sized opaque envelope in their mailbox. The envelope had a window through which it could be seen that the letter was addressed to the inhabitant of the house. A note of €5 could be seen through this window as well, to give subjects an incentive to open the envelope and read its contents. The envelope contained a set of instructions, as well as a small stamped envelope with a card. The instructions explained that the subject was selected randomly for participation in a scientific study of Erasmus University Rotterdam, and that the study aimed to investigate the use of

the postal services in various neighborhoods in the Netherlands. Subjects were instructed to mail the attached stamped envelope to the University's mail address. If the card was received within four weeks time, then €20 would be paid. Participation numbers were used in the study to ensure anonymity.

In the instruction letter, mention was made of the principal investigator, along with his contact information and a website that contained more details on the study (see below). The Private Incentive treatment was conducted on one single day in October 2014. To test for noise that could be introduced in our data due to the mail company, we posted ourselves seventeen envelopes from this treatment directly to the university's mail address. These envelopes were sent from the neighborhoods that the subjects live in. As expected, all of these envelopes arrived the day after sending them out. We conclude that our data are free of any noise due to the mail company.

Instructions (translated from Dutch)

To the inhabitants of [household address]

Rotterdam, 14 September 2014

Subject: Earn €20 by participating in a scientific study

Dear sir/madam,

You have been randomly selected for participation in a **scientific study** of **Erasmus University Rotterdam**. We are studying the use of the Dutch postal services in different neighborhoods across the Netherlands. To show you that this is a serious study and compensate you for your time, we have **enclosed €5**.

Will I get paid for my participation? Yes you will! In addition, to the enclosed €5 which is yours to keep, if you complete the study, we will send you an **additional €20 in cash**.

What do I have to do? It is really easy to complete this study. All you need to do is put the card in the enclosed envelope, and then **mail the envelope**. If you complete the study by **October 3**, we will send you the additional €20, which you should receive by October 17. If you choose not to mail the envelope, then you will not complete the study, and you will not receive the €20 completion payment. You may naturally keep the enclosed €5 whether you complete the study or not.

Will my personal information be revealed? No, it will not. You will notice that the enclosed card does not have your name on it, but only a participant number. This participant number links the card to your address, so that we can send your €20 for completing the study to your address. We do not record your name. Your participation therefore will be anonymous.

Thank you very much for participating in this research.

Best regards,

Prof. J.T.R. Stoop

Flyer that accompanied the instructions

More information about this study

This study is conducted by Erasmus University Rotterdam, led by Prof J.T.R. Stoop. You can learn about Prof. Stoop on the Internet at

http://people.few.eur.nl/stoop/[2]

After March 31, 2015 you can or learn more about this study at

http://people.few.eur.nl/stoop/postalstudy.htm

If you have questions, please contact:

Prof. J.T.R. Stoop, Dutch Postal Study
Department of Applied Economics
Erasmus School of Economics, PO Box 1738
3000 DR Rotterdam, the Netherlands
Tel.: +31 (0)10 408 27 08
Email: stoop@ese.eur.nl

Thank you very much for participating in this research.

Accompanying text on the internet website (during data gathering process)

THE ERASMUS UNIVERSITY POSTAL STUDY

Lead investigator:

Prof. J.T.R. Stoop
Department of Applied Economics
Erasmus School of Economics, PO Box 1738
3000 DR Rotterdam, the Netherlands
Tel.: +31 (0)10 408 27 08
Email: stoop@ese.eur.nl

Purpose of the Research:

[2] It is important to note that, at the time of running the Private Incentives treatment, there was no information on Prof. Stoop's website that could alert respondents about the research question or our hypotheses.

The Erasmus University Rotterdam Postal Study (EURPS) is examining how people in different parts of the Netherlands use the postal service and send letters.

Funding for this Research:

This study is supported by grants from the Erasmus School of Economics and the Netherlands organisation for Scientific Research.

Privacy Statement:

The privacy of participants in this research is assured. All addresses used in this study are randomly selected. As is required by research ethics, participants' names are never known to the EURPS researchers, and after the data collection EURPS will destroy any record of the addresses used. There will be no information in our data what will allow us to identify any of the participants. In addition, our report will not mention any specific parts of Netherlands. The data will not be shared with anyone outside of the research group, and will be used only for scientific purposes.

Important dates:

October 1, 2013: Data Collection Begins.
December 31, 2014: Data Collection Ends.
March 31, 2015: Report on the Results of the Data Collection will be publicly available and posted on this Web Site.

Please check back after March 31, 2015 for more details about this study.

Thank you very much.

Accompanying text on the internet website (after data gathering process)

THE ERASMUS UNIVERSITY POSTAL STUDY
Lead investigator:

Prof. J.T.R. Stoop
Department of Applied Economics
Erasmus School of Economics, PO Box 1738
3000 DR Rotterdam, the Netherlands
Tel.: +31 (0)10 408 27 08
Email: stoop@ese.eur.nl

Purpose of the Research:

We are studying how people in different parts of the Netherlands use the postal service. We have randomly selected households to participate in the study. For their effort, €5 was

included. The task required subjects to send a card that was given to them. If the card was received, then €20 was returned in cash. The results are interesting to us, because they shed light on the level of compensation needed for people to take the effort to mail a letter.

References

[1] Joseph Henrich, Richard Boyd, Samuel Bowles, Colin Camerer, Ernst Fehr, and Herbert Gintis. *Foundations of Human Sociality: Economic Experiments and Ethnographic Evidence from Fifteen Small-Scale Societies.* Oxford University Press, New York, 2004.

[2] Benedikt Herrmann, Christian Thöni, and Simon Gächter. Antisocial punishment across societies. *Science*, 319:1362–1367, March 2008.

[3] Nancy R Buchan, Eric J Johnson, and Rachel TA Croson. Let's get personal: An international examination of the influence of communication, culture and social distance on other regarding preferences. *J. Econ. Behav. Organ.*, 60(3):373–398, 2006.

[4] Gary Charness, Ernan Haruvy, and Doron Sonsino. Social distance and reciprocity: An internet experiment. *J. Econ. Behav. Organ.*, 63(1):88–103, 2007.

[5] Paul Smeets, Ashley Whillans, Rene Bekkers, and Michael I Norton. Time use and happiness of millionaires: Evidence from the netherlands. *Soc. Psychol. Pers. Sci.*, 11(3):295–307, 2019.
