# Supplementary material for: Higher socioeconomic status does not predict decreased prosocial behavior in a field experiment
Source: Nat Commun. 2021 Jul 12;12:4266. doi: 10.1038/s41467-021-24519-5 (PMC8275767; doi:10.1038/s41467-021-24519-5)
Supplement: Supplementary file 3 — Description of Additional Supplementary Files [file 41467_2021_24519_MOESM3_ESM.pdf]

Description of Additional Supplementary Files

File Name: Supplementary Data 1
Description: Includes the data from the main experiment (misdelivered envelopes)

File Name: Supplementary Data 2
Description: Includes the survey data exploring individuals' knowledge on how to return misdelivered envelopes.

File Name: Supplementary Data 3
Description: Includes the survey data concerning attitudes toward postal and other public services.

File Name: Supplementary Code 1
Description: This file contains the statistical code used for the analysis of our data.
